# Supplementary material for: How health securitisation shapes health system priorities: A realist synthesis
Source: PLOS Glob Public Health. 2025 May 28;5(5):e0004677. doi: 10.1371/journal.pgph.0004677 (PMC12118837; doi:10.1371/journal.pgph.0004677)
Supplement: S1 Data — (DOCX) [file pgph.0004677.s001.docx]

S1 Data - Findings

1. Kelly AH, Lezaun J, Löwy I, Matta GC, De Oliveira Nogueira C, Rabello ET. Uncertainty in times of medical emergency: Knowledge gaps and structural ignorance during the Brazilian Zika crisis. Soc Sci Med [Internet]. 2020 Feb [cited 2024 Apr 17];246:112787. Available from: <https://linkinghub.elsevier.com/retrieve/pii/S027795362030006X>
2. Wenham C, Farias DB. Securitizing Zika: The case of Brazil. Secur Dialogue [Internet]. 2019 Oct 1 [cited 2023 Jun 29];50(5):398–415. Available from: <https://doi.org/10.1177/0967010619856458>
3. Fehross A, Pahlman K, Silva DS. Ethics and Health Security in the Australian COVID-19 Context: A Critical Interpretive Literature Review. J Bioethical Inq [Internet]. 2023 Nov 8 [cited 2023 Nov 29]; Available from: https://link.springer.com/10.1007/s11673-023-10255-6
4. Kennedy D. Global health governance responds to COVID-19: Does the security/access divide persist? Glob Public Health [Internet]. 2023 Jan 2 [cited 2023 Dec 14];18(1):2200296. Available from: https://www.tandfonline.com/doi/full/10.1080/17441692.2023.2200296
5. Wenham C, Abagaro C, Arévalo A, Coast E, Corrêa S, Cuéllar K, et al. Analysing the intersection between health emergencies and abortion during Zika in Brazil, El Salvador and Colombia. Soc Sci Med [Internet]. 2021 Feb [cited 2024 Jan 17];270:113671. Available from: https://linkinghub.elsevier.com/retrieve/pii/S0277953621000034
6. Wenham C, Arauz-Reyes NM, Meneses-Sala D, Rueda-Borrero C. Explicitly sexing health security: analysing the downstream effects of Panama’s sex-segregated COVID-19 disease control policy. Health Policy Plan [Internet]. 2022 Jun 13 [cited 2024 Jan 30];37(6):728–36. Available from: https://academic.oup.com/heapol/article/37/6/728/6516119
7. Kamradt-Scott A, McInnes C. The securitisation of pandemic influenza: Framing, security and public policy. Glob Public Health [Internet]. 2012 Dec 1 [cited 2023 May 3];7(sup2):S95–110. Available from: https://doi.org/10.1080/17441692.2012.725752
8. Kolie D, Delamou A, Van De Pas R, Dioubate N, Bouedouno P, Beavogui AH, et al. ‘Never let a crisis go to waste’: post-Ebola agenda-setting for health system strengthening in Guinea. BMJ Glob Health [Internet]. 2019 Dec [cited 2024 Jan 18];4(6):e001925. Available from: https://gh.bmj.com/lookup/doi/10.1136/bmjgh-2019-001925
9. Kirk J, McDonald M. The Politics of Exceptionalism: Securitization and COVID-19. Glob Stud Q [Internet]. 2021 Sep 1 [cited 2024 Mar 4];1(3):ksab024. Available from: https://doi.org/10.1093/isagsq/ksab024
10. Metelmann IB, Flessa S, Busemann A. Does health securitization affect the role of global surgery? J Public Health [Internet]. 2022 Apr [cited 2024 Jan 18];30(4):925–30. Available from: https://link.springer.com/10.1007/s10389-020-01347-3
11. Holst J. Global Health – emergence, hegemonic trends and biomedical reductionism. Glob Health [Internet]. 2020 Dec [cited 2024 Mar 4];16(1):42. Available from: https://globalizationandhealth.biomedcentral.com/articles/10.1186/s12992-020-00573-4
12. Taylor S. In pursuit of zero: Polio, global health security and the politics of eradication in Peshawar, Pakistan. Geoforum [Internet]. 2016 Feb [cited 2024 Jan 29];69:106–16. Available from: https://linkinghub.elsevier.com/retrieve/pii/S0016718515302578
13. Davis M. Compliant, complacent or panicked? Investigating the problematisation of the Australian general public in pandemic influenza control. Soc Sci. 2011;
14. Benton A. 2 Whose Security? Militarization and Securitization During West Africa’s Ebola Outbreak .
15. Vearey J, de Gruchy T, Maple N. Global health (security), immigration governance and Covid-19 in South(ern) Africa: An evolving research agenda. J Migr Health [Internet]. 2021;3. Available from: https://www.scopus.com/inward/record.uri?eid=2-s2.0-85111539858&doi=10.1016%2fj.jmh.2021.100040&partnerID=40&md5=af036ae6e77c88cd16788330d0373ed8
16. Holst J, van de Pas R. The biomedical securitization of global health. Glob Health [Internet]. 2023 Mar 4 [cited 2023 Apr 25];19:15. Available from: https://www.ncbi.nlm.nih.gov/pmc/articles/PMC9985490/
17. Jackson C, Habibi R, Forman L, Silva DS, Smith MJ. Between rules and resistance: moving public health emergency responses beyond fear, racism and greed. BMJ Glob Health [Internet]. 2022 Dec 5 [cited 2023 Aug 16];7(12):e009945. Available from: https://www.ncbi.nlm.nih.gov/pmc/articles/PMC9723907/
18. Russell EK, Phillips T, Gaylor A, Trabsky M. ‘It is not about punishment, it’s about protection’: Policing ‘vulnerabilities’ and the securitisation of public health in the COVID-19 pandemic. Criminol Crim Justice [Internet]. 2022 Aug 31 [cited 2024 Jan 9];174889582211204. Available from: http://journals.sagepub.com/doi/10.1177/17488958221120480
19. Parker M, Baluku M, Ozunga BE, Okello B, Kermundu P, Akello G, et al. Epidemics and the Military: Responding to COVID-19 in Uganda. Soc Sci Med [Internet]. 2022 Dec [cited 2023 Apr 14];314:115482. Available from: https://linkinghub.elsevier.com/retrieve/pii/S0277953622007882
20. Boland ST, Mayhew S, Balabanova D. Securitising public health emergencies: a qualitative examination of the origins of military intervention in Sierra Leone’s Ebola Epidemic. BMJ Public Health [Internet]. 2023 Aug [cited 2023 Nov 20];1(1):e000236. Available from: https://bmjpublichealth.bmj.com/lookup/doi/10.1136/bmjph-2023-000236
21. Watterson C, Kamradt-Scott A. Fighting Flu: Securitization and the Military Role in Combating Influenza. Armed Forces Soc [Internet]. 2016 Jan 1 [cited 2024 Jan 15];42(1):145–68. Available from: https://doi.org/10.1177/0095327X14567364
22. Østebø MT, Østebø T, Tronvoll K. Health and politics in pandemic times: COVID-19 responses in Ethiopia. Health Policy Plan [Internet]. 2021 Dec 1 [cited 2024 Jan 18];36(10):1681–9. Available from: https://doi.org/10.1093/heapol/czab091
23. Rushton S. Framing AIDS: Securitization, Development-ization, Rights-ization.
24. Hecita IJR, Torneo AR, Panelo DE, Miranda K. 5. Securitisation of the pandemic response in the Philippines. Reg Stud Policy Impact Books [Internet]. 2023 Jan 2 [cited 2023 Jun 29];5(1):89–109. Available from: https://doi.org/10.1080/2578711X.2023.2196215
25. Hapal K. The Philippines’ COVID-19 Response: Securitising the Pandemic and Disciplining the Pasaway. J Curr Southeast Asian Aff [Internet]. 2021 Aug 1 [cited 2023 Jun 29];40(2):224–44. Available from: https://doi.org/10.1177/1868103421994261
26. Chung R. The securitization of health in the context of the war on terror. National security and global health: the conflict of imperatives. Med Confl Surviv [Internet]. 2017 Jan 2 [cited 2023 Apr 27];33(1):32–40. Available from: https://doi.org/10.1080/13623699.2017.1327150
27. Barker K. Infectious Insecurities: H1N1 and the politics of emerging infectious disease. Health Place [Internet]. 2012 Jul [cited 2024 Feb 9];18(4):695–700. Available from: https://linkinghub.elsevier.com/retrieve/pii/S1353829212000056
28. Bengtsson L, Rhinard M. Securitisation across borders: the case of ‘health security’ cooperation in the European Union. West Eur Polit [Internet]. 2019 Feb 23 [cited 2024 Mar 4];42(2):346–68. Available from: https://doi.org/10.1080/01402382.2018.1510198
29. Elbe S. Haggling over viruses: The downside risks of securitizing infectious disease. Health Policy Plan [Internet]. 2010;25(6):476–85. Available from: https://www.scopus.com/inward/record.uri?eid=2-s2.0-77958601020&doi=10.1093%2fheapol%2fczq050&partnerID=40&md5=258588e6be7c5166d3063d34f6619cc4
30. Wishnick E. Dilemmas of securitization and health risk management in the People’s Republic of China: the cases of SARS and avian influenza. Health Policy Plan [Internet]. 2010 Nov 1 [cited 2023 Sep 28];25(6):454–66. Available from: https://academic.oup.com/heapol/article-lookup/doi/10.1093/heapol/czq065
31. Lo Yuk-ping C, Thomas N. How is health a security issue? Politics, responses and issues. Health Policy Plan [Internet]. 2010 Nov 1 [cited 2023 Sep 21];25(6):447–53. Available from: https://doi.org/10.1093/heapol/czq063
32. Goldizen FC. From SARS to Avian Influenza: The Role of International Factors in China’s Approach to Infectious Disease Control. Ann Glob Health [Internet]. 2016 [cited 2024 Mar 4];82(1):180–8. Available from: https://www.ncbi.nlm.nih.gov/pmc/articles/PMC7103944/
33. Horner J, Wood JG, Kelly A. Public health in/as ‘national security’: tuberculosis and the contemporary regime of border control in Australia. Crit Public Health [Internet]. 2013 Dec 1 [cited 2024 Apr 1];23(4):418–31. Available from: https://doi.org/10.1080/09581596.2013.824068
34. Mhazo AT, Maponga CC. Governing a pandemic: biopower and the COVID-19 response in Zimbabwe. BMJ Glob Health [Internet]. 2022 Dec 1 [cited 2023 Apr 24];7(12):e009667. Available from: https://gh.bmj.com/content/7/12/e009667
35. Dimari G, Papadakis N. The securitization of the Covid-19 pandemic in Greece: a just or unjust securitization? Qual Quant [Internet]. 2023 [cited 2023 Apr 27];57(Suppl 1):77–97. Available from: https://www.ncbi.nlm.nih.gov/pmc/articles/PMC8853285/
36. Honigsbaum M. Between Securitisation and Neglect: Managing Ebola at the Borders of Global Health. Med Hist [Internet]. 2017 Apr [cited 2023 Jul 11];61(2):270–94. Available from: https://www.cambridge.org/core/journals/medical-history/article/between-securitisation-and-neglect-managing-ebola-at-the-borders-of-global-health/4DA04DA13C96C2A2589ED2380739F904
37. Obasa AE, Singh S, Chivunze E, Burgess T, Masiye F, Mtande T, et al. Comparative strategic approaches to COVID-19 in Africa: Balancing public interest with civil liberties. South Afr Med J Suid-Afr Tydskr Vir Geneeskd [Internet]. 2020 Aug 13 [cited 2023 Dec 14];110(9):858–63. Available from: https://www.ncbi.nlm.nih.gov/pmc/articles/PMC8066401/
38. Kuteleva A, Clifford SJ. Gendered securitisation: Trump’s and Putin’s discursive politics of the COVID-19 pandemic. Eur J Int Secur [Internet]. [cited 2024 Mar 11];1–17. Available from: https://www.ncbi.nlm.nih.gov/pmc/articles/PMC8007944/
39. Smith J. Overcoming the ‘tyranny of the urgent’: integrating gender into disease outbreak preparedness and response. Gend Dev [Internet]. 2019 May 4 [cited 2024 Jan 17];27(2):355–69. Available from: https://www.tandfonline.com/doi/full/10.1080/13552074.2019.1615288
40. Elbe S, Voelkner N. Viral sovereignty: The downside risks of securitizing infectious disease. In: The Handbook of Global Health Policy [Internet]. 2014. p. 305–17. Available from: https://www.scopus.com/inward/record.uri?eid=2-s2.0-85083900541&doi=10.1002%2f9781118509623.ch16&partnerID=40&md5=3184cab38f8f96d63e9c72076ba77af8
41. Karyotis G, Connolly J, Collignon S, Judge A, Makropoulos I, Skleparis D. What Drives Support for Social Distancing? Pandemic Politics, Securitisation and Crisis Management in Britain.
42. Stott C, West O, Harrison M. A Turning Point, Securitization, and Policing in the Context of Covid-19: Building a New Social Contract Between State and Nation? Polic J Policy Pract [Internet]. 2020 Apr 29 [cited 2024 Jan 30];paaa021. Available from: https://www.ncbi.nlm.nih.gov/pmc/articles/PMC7197571/
43. Candelaria JL, Talamayan F. Civil–Military Relations and the Securitisation of the COVID-19 Crisis in the Philippines and Malaysia. In: Shukri S, editor. Pandemic, Politics, and a Fairer Society in Southeast Asia: A Malaysian Perspective [Internet]. Emerald Publishing Limited; 2023 [cited 2024 Jan 15]. p. 53–65. Available from: https://www.emerald.com/insight/content/doi/10.1108/978-1-80455-588-020231005/full/html
44. Herington J. Securitization of infectious diseases in Vietnam: the cases of HIV and avian influenza. Health Policy Plan [Internet]. 2010 Nov 1 [cited 2024 Feb 12];25(6):467–75. Available from: https://academic.oup.com/heapol/article-lookup/doi/10.1093/heapol/czq052
45. Hanrieder T, Kreuder-Sonnen C. WHO decides on the exception? Securitization and emergency governance in global health. Secur Dialogue [Internet]. 2014 Aug [cited 2024 Feb 12];45(4):331–48. Available from: http://journals.sagepub.com/doi/10.1177/0967010614535833
46. Kentikelenis A, Seabrooke L, Sending OJ. Global Health Expertise in the Shadow of Hegemony. Stud Comp Int Dev [Internet]. 2023 Sep 1 [cited 2024 Apr 2];58(3):347–68. Available from: https://doi.org/10.1007/s12116-023-09405-z
47. Kaunert C, Leonard S, Wertman O. Securitization of COVID-19 as a Security Norm: WHO Norm Entrepreneurship and Norm Cascading. Soc Sci [Internet]. 2022 Jun 21 [cited 2023 Apr 14];11(7):266. Available from: https://www.mdpi.com/2076-0760/11/7/266
48. Voss M, Kump I, Bochtler P. Unpacking the framing of health in the United Nations Security Council. Aust J Int Aff [Internet]. 2022 Jan 2 [cited 2024 Apr 1];76(1):4–10. Available from: https://doi.org/10.1080/10357718.2021.2017845
49. McCoy D, Roberts S, Daoudi S, Kennedy J. Global health security and the health-security nexus: principles, politics and praxis. BMJ Glob Health [Internet]. 2023 Sep [cited 2023 Nov 21];8(9):e013067. Available from: https://gh.bmj.com/lookup/doi/10.1136/bmjgh-2023-013067
50. Baringer L, Heitkamp S. Securitizing Global Health: A View from Maternal Health.
51. Wenham C. The oversecuritization of global health: changing the terms of debate. Int Aff [Internet]. 2019 Sep 1 [cited 2023 Jul 27];95(5):1093–110. Available from: https://doi.org/10.1093/ia/iiz170
