# Supplementary material for: How health securitisation shapes health system priorities: A realist synthesis
Source: PLOS Glob Public Health. 2025 May 28;5(5):e0004677. doi: 10.1371/journal.pgph.0004677 (PMC12118837; doi:10.1371/journal.pgph.0004677)
Supplement: Attachment — Submitted filename: Response to reviewers - PGPH-D-24-02972 .docx [file pgph.0004677.s003.docx]

**PGPH-D-24-02972
How health securitisation shapes health system priorities: a realist synthesis
PLOS Global Public Health**

All responses to reviewers begin with two asterisks **. Please note that the Lines referred to in author responses are lines as per the unmarked manuscript.

**Review Comments to the Author**

**Reviewer #1**: Overall, I think this is a great paper: well written, based on rigorous analysis, on an important topic.

However, I do have a few comments, of which I hope they can be addressed in a review. Before that, some positionality reflections are in order: I am one of those people from the Global North, mentioned in lines 782-792, who use ‘securitisation’ as a means to mobilise international financial transfers for health. It is not an argument I like, but it may be the only argument that works. By the way, I also use it to argue for a more balanced global health governance.
So here are my comments.

** We thank the reviewer for their kind assessment of the work and appreciate the positionality reflections.

1. You use realist evaluation, describing and analysing context-mechanism-outcomes of securitisation. But the focus of the paper is on mechanisms: the contexts are summarized and very little is written about outcomes. Without having read the papers on which the analysis is based, I do not know if the outcomes mentioned in figure 4 are real outcomes or desired outcomes. Would it be possible to clarify that?

**We thank the reviewer for the feedback, and we take this on board. We have now made it clear at the beginning of the findings section, that the outcomes mentioned in Figure 4 are real outcomes described or inferred in – and extracted from – the papers included in the review. Please see L300-304

2. I am a bit surprised that ‘common interests’ was not identified as a mechanism. Slogans like “No one is safe, until we are all safe” <https://www.afro.who.int/sites/default/files/Progress%20report%2021/docs/WHO-AFRO_SPRP_COVID-19_DG-Message.pdf> (and papers using that logic) can indeed be considered as a call to self-protection via action abroad, but a more benign interpretation would be that it is a call to behave as ‘citizens of the world’ or something like that. Did that not come out at all or did you choose to put that under ‘self-protection’?

**We thank the reviewer for this feedback. We are aware of slogans as “no one is safe until we are all safe”. We chose to put that category of rationale under ‘self-protection’ – interpreted as a call to self-protect through action abroad (or elsewhere, or on other groups) is indeed in the act of self-interest; the idea that while everyone may be called upon as citizens or participants or concerned parties of the "securitisation" efforts, everyone may not be protected equally by those efforts, and some may bear a heavier burden of the measures necessary for securitisation than others.

3. With regards to the ‘norm-setting’ mechanism, I think it is important to make a clearer distinction between norm-setting itself and encouraging norm compliance. The International Health Regulations, for example, are international law: the norms are set at the negotiation tables in Geneva. I have not read the papers that are mentioned here (endnotes 45-49) but looking at the titles, they seem more about norm compliance than about norm setting.

** We thank the reviewer for this comment and agree with the reviewer on the need for a clearer distinction. We conceptualised the act of norm-setting to include also as an act of norm-compliance, i.e., norm-setting in compliance with another set of norms that are set elsewhere – responding to and complying with certain norms, and with it, setting the norms in a different place. We have now included this in the manuscript for clarity. Please see lines 595-601

**Reviewer #2**: Summary:
This is a well-written article authored by several global health and health security thought leaders. It explores the mechanisms leading to the securitization of health and health systems (i.e., health security). The authors cogently build upon existing theory—namely the Copenhagen School’s Securitisation Theory—to expand the literature in substantive and useful ways, and identify five mechanisms—uncertainty, self-protection, self-reliance, self-preservation, and norm-setting—by which ‘health security’ framings are adopted. The manuscript represents a work that I anticipate will draw significant interest from a wide audience across several public health subfields. There are, however, several major and minor points that should be addressed before its publication.

** We thank the reviewer for this assessment of the work and appreciate the feedback about its potential to generate interest.

Major Issues:
Methods, P4, L134 – The omission of ‘epidemic’ as a search is curious, especially considering that most health security events since 2010 (e.g., Ebola, polio, Zika, mpox) have not been characterized by the WHO as a pandemic, but rather ‘epidemics’ or ‘global epidemics.’ I recognize that this might seem like splitting hairs, but I strongly encourage the authors to consider running another search with ‘epidemic’ to ensure that they did not inadvertently exclude any relevant literature in their review.

** We thank the reviewer for this comment and appreciate that the examples provided were all classified as epidemics under health security events. The literature identified in our search and included in our review were reports of all such events, despite not using ‘epidemic’ in our search. We are mindful that typically, "outbreak carries the same definition of epidemic, and it is hardly conceivable that the word "epidemic" is used in an article without requiring the word "outbreak" to say what it means. As realist syntheses do not aim to be exhaustive in their identification of the literature nor produce reproducible results but aim instead to develop, refine, or test theories in a way that generates explanatory insights, we believe that while unlikely, a potential omission of some articles will not be detrimental to the causal insights reported in this review. We also cannot imagine how such an omission (if real) could lead to a systematic bias in the sample of articles we have included in the synthesis.

Methods, P4, L149 – The authors state that engaging with the concept of ‘securitisation’ was among the inclusion/exclusion criteria but never define how ‘security’ or ‘securitisation’ was conceptualized in their study. This needs to be done to promote transparency—especially considering that, in their own words, realist syntheses ‘are not reproducible or standardized.’ The authors should, at a minimum, strive to leave readers with a better understanding of how they conceptualized security to promote comprehension of what they did and why certain articles were included and/or excluded.

** We thank the reviewer for this feedback and appreciate why such a clarification is required. We have, therefore, edited the section to reflect the suggestion – i.e., how securitisation has been conceptualised. Please see lines 221-233

Results/Discussion – The authors note that they consider the international, national, and subnational levels (P3, L103), but there is relatively little discussion of the subnational level. I recommend that they revise the manuscript to include more in-depth discussion of how these mechanisms operate at subnational levels or revise the manuscript to narrow the scope to the international and national levels and keep the bits in the Discussion section (e.g., ‘dynamics at the national-subnational interface’).
- go back and revise/bring our sub-national

** We thank the reviewer for this comment and appreciate the need to have more in-depth inclusion of mechanisms at the sub-national level. The mechanisms were indeed identified such that they could work at the national/subnational interface as much as the international/national interface. Indeed, in some cases, data included in the analysis presented in the results section came from articles that discussed national/subnational governance, though this was not always made explicit. We agree that making it explicit in the results and discussion sections is important to, at minimum make a case for why such a dynamic is equally important. We have now edited certain sections of the manuscript to emphasise these points. Please see Lines: 370-371, 503-504. 528-531, 661-678, 752-756, 804-808

Results/Discussion – The authors note that one limitation of their realist synthesis is that it is overly critical of health security framings. Recognizing that the origins of health security are rooted in colonial and military origins and that the past several years have witnessed growing literature and advocacy efforts focused on decolonizing global health, this critical lens is unsurprising. There is value and validity in this literature and these efforts and health security must grapple with its less than honorable origins. The authors do a very good job of highlighting this. Still, while the authors cannot change the articles identified and included in the review, the manuscript is in essence a narrative and currently reads as a very biased one. Consider, for example, the discussion of military force in the Self-Protection section. While there are certainly power dynamics at play between Western and non-Western countries, Western countries are not deploying their militaries to respond to a health emergency in a foreign country without the explicit permission of said country. To do so would be an egregious violation of sovereignty and an act of war. And, while the precise motivations for engaging with public health responses in this way may be obscure, they are surely not entirely rooted in self-preservation. For instance, thousands of military personnel were deployed from Canada, China, France, Germany, the USA, and the UK to support the 2014 Ebola response—a disease that while highly lethal, is not especially contagious. It seems highly unlikely that all of these countries would have been motivated to send their militaries to support an outbreak response for a disease that is not especially contagious (and, therefore, relatively unlikely to pose existential threats to their respective country) and likely less lethal in better resourced settings (and, therefore, not as serious of a threat) in the name of ‘self-preservation.’

** We thank the reviewer for this very important comment and appreciate the insights provided. We agree that the deployment and involvement of foreign military and aid during Ebola outbreak was in many parts unjustifiable – given, as the reviewer points out, that it would likely be curtailed in a better resource environment. However, the narrative during the Ebola outbreak was that it was indeed highly contagious and highly lethal (as the reviewer points out), and the narrative was one of ‘protecting global health security’ (2014 GHSA was developed), even if Ebola did not pose an existential threat in a traditional sense. Indeed, securitisation does not require or imply an objective or immediate existential threat. A health issue only needs to be so framed. Securitisation is in the framing and the response. Ebola did not pose such an existential threat but it became the interest of powerful countries once a case was exported beyond the West African borders. Even if Western countries had permission to deploy troops (which they could obtain if they wanted), their involvement could still be shaped by self-interest/protection—whether in terms of geopolitical influence or reinforcing global (health) governance structures that favour them. It has been argued that from a ‘Western perspective’ without military assistance, the outbreak could have exacerbated political and economic instability, potentially leading to state failure & regional conflict (including, perhaps fear of mass migration to their own countries). We have now made this point in the discussion. Please see lines 735-742.

Another example comes in the authors’ discussion of a ‘Western nation ‘at war.’ The focus on race is one manifestation, but far from the only, form of this stigmatization. For example, consider how certain sexually transmissible diseases have led to the stigmatization of minority gender groups within Western nations (e.g., HIV, mpox, etc.). Ultimately, I would challenge the authors to provide a much more thoughtful and balanced discussion in the Results and/or Discussion section because, as is, the manuscript currently begs the question of ‘why does the health security framing exist at all?’ And, despite its flaws and unsavory origins, there is, surely, some value in the framing or else it would not be used.

** We thank the reviewer once again for this thoughtful feedback and insights. The ‘western nation at war’ example in the findings sections under self-protection was an example/outcome extracted from an article included in the review, and we appreciate that there are indeed other forms of stigmatisation that may encourage securitisation or be a result of securitised responses. For example, in lines 346-350 the impact on the transgender community (as an outcome) has been highlighted under uncertainty. The securitisation and de-securitisation of mpox has also been discussed – lines 768-785 – although not explicitly in relation to the stigmatisation of sexually transmitted diseases. The challenge to be more balanced is an important one, for which we are grateful. We consider the challenge to "why securitisation?" to be similar to the challenge of "why stigmatization?" That is: “Is stigmatisation (or securitisation) sometimes necessary? Surely there is some value in stigmatisation (or securitisation), else it would not be used.” In other words, it is possible to argue that even though stigmatisation comes from a place of prejudice, perhaps it has some real or potential value in that it may help limit the spread of an infectious disease. Our response to this challenge, as might be the reviewer's response too, and which was the response suggested by the literature which we have synthesised, was that: there are perhaps other ways to limit the spread of an infectious disease that do not involve stigmatisation.

These were the thoughts that went through our mind as we considered the reviewer's challenge. Our conclusion was that a measure may be used because it is of value to some groups/governments, especially if the group to whom that measure is of value are not the group that gets to suffer the burden of the measure. We agree that there are arguments that could be made on behalf of securitisation, but we are concerned such arguments are akin to those that may be made on behalf of stigmatisation.

Minor Issues:
Introduction, P3, L104 – The authors provide some context for the ‘realist approach to evidence synthesis’ in the Methods section, but I recommend also adding a brief sentence explaining what the ‘realist approach,’ in the Introduction ideally before the sentence describing what it is well-suited to answer. It’s nice to know that the approach is suited for answering ‘what works, for whom, in what context?’ but if readers are unfamiliar with the method, they are likely to find this statement confusing.

** We thank the reviewer for this insight, and we agree that such a clarification is needed. We have now edited to reflect this please L104-111

Introduction, P3, L105 – Change ‘question’ to ‘questions’ as there are multiple posed.

** We thank the reviewer for this – we have now edited to reflect this please see line 123

Methods, P4, L170 – The authors should provide more detail about the richness and rigour assessments. What did these assessments entail? Were there criteria used to systematically evaluate the richness and rigour of articles?

** We thank the reviewer for this suggestion. The richness and rigour assessment in a realist synthesis primarily relies on the researcher’s experiences (reflected in the choice of "program theory" used to make sense of a phenomenon) and assessment (of the extent to which a particular piece of evidence or insight helped to illuminate, challenge, quality, extend, or refute the "program theory") – we have clarified this and provided additional information -please see lines 246-254

Methods, P6, Line210 – The Theoretical Framing subsection is very useful for understanding the manuscript and answered many questions I had when reading the article for the first time. I would recommend the authors consider moving the bulk of this, or at least the first 2 paragraphs, to the beginning of the Methods section.

** We appreciate this thoughtful feedback, and we have now moved the Theoretical Framing section to the beginning of the Methods section, as suggested. Lines 128 onwards.

Methods, P7, Line254 – Recommend defining ‘C-M-O’ in the text to facilitate reading.

** We thank the reviewer for picking this up – we have now defined the CMO in text – line 172

Results, P8, Line298 – Revise ‘countries’ to ‘geographies’ or remove Sao Paulo from the list (as it is not a country).

** We thank the reviewer for picking up this error, we have now changed Sao Paulo to Brazil, in keeping with countries names – line 293-298

Results, P15, Line621 – The JEE is but one tool in a broader IHR M&E framework. The other elements—including the SPAR and NAPHS process—also seem relevant for norm-setting. Recommend that the authors mention these, as well.

** We thank the reviewer for this comment, and we have now included frameworks such as SPAR & NAPHS as an example of norm-setting. Please see lines 632-939

Results, P8–16 – Noting the reference style in the manuscript changes.

** We appreciate the reviewer picking this up – in line 190-191 we have referred to the reference style change – in the results/findings section lines 377-690, we use superscripts to refer to the article where we derived the insights for each part of a C-M-O as to refer to the actual articles where data was extracted.

Results, P8 – The uncertainty mechanism seems particularly relevant for emerging infectious diseases (e.g., COVID-19) or for reemerging diseases in new geographies (e.g., Zika). The authors should highlight this because it contributes, at least in part, to why certain disease outbreaks and public health emergencies are not securitized by national and international actors.

** We thank the reviewer for this comment, and agree with this insight, and have highlighted this in lines 726-730

Results – The focus on post-civil war environments is interesting, but I wonder if the authors would consider revising this focus to ‘areas of civil unrest’ or something similar. To me, it seems equally plausible that many of the discussed outcomes could also come before a civil war or in an area experiencing widespread civil strife.

**We appreciate this feedback and agree that the context of civil unrest that goes beyond the post-civil war environment is plausible for similar outcomes. We have now revised to reflect this change. Please see lines 405-410, 515

Figure 3 – The authors should consider providing a new title. The current one is not terribly helpful for understanding what is being depicted in the figure.

** We thank the reviewer for this suggestion. We have now changed the title of the graph to “Pathways to securitised responses: mechanisms by which ‘health security’ framing is adopted in response to an event”

Figure 4 – This is the article in figure form, and it has immense potential. It is what will be used lecture slides. It is what will be used in policy briefs. And it is, currently, nearly illegible because there is far too much text. The authors should work to reduce how busy this figure is. For example, “Pre-pandemic/emergency environment in which governments are pressured or encouraged to activate a whole-government response to a crisis’ could be shortened to simply read ‘Governments are pressured or encouraged to activate a whole-government response.’ I would encourage the authors to try to shorten text to one line, maximum.

**We thank the reviewer for the thoughtful feedback and have amended the figure to ensure legibility and ease of comprehension.

Please see the new figure -line 691
